# The Interaction of SRL-2 Peptide with LRP-1 Receptor and Identification of Breast Cancer Related Biomarkers: An In-silico Approach

**DOI:** 10.5812/ijpr-136624

**Published:** 2023-06-11

**Authors:** Yasamin Davatgaran-Taghipour, Yousof Saeedi-Honar, Roya Salehi, Amir Zarebkohan, Vladimir P.Torchilin

**Affiliations:** 1 Student Research Committee, Tabriz University of Medical Sciences, Tabriz, Iran; 2 Department of Medical Nanotechnology, School of Advanced Medical Sciences, Tabriz University of Medical Sciences, Tabriz, Iran; 3 Department of Life Sciences and Biotechnology, Shahid Beheshti University, Tehran, Iran; 4 Drug Applied Research Center, Tabriz University of Medical Sciences, Tabriz, Iran; 5 Center for Pharmaceutical Biotechnology and Nanomedicine and Department of Chemical Engineering, Northeastern University, Boston, USA

**Keywords:** Breast Cancer, Chimeric Peptide, LRP-1, Meta-analysis, Biomarkers

## Abstract

**Background:**

Breast cancer is a multifaceted disease characterized by genetic and epigenetic changes that lead to uncontrolled cell growth and metastasis. Early detection and treatment are crucial for managing diseases.

**Objectives:**

The objective of this study is to investigate the potential of chimeric peptides for drug delivery and to identify biomarkers associated with breast cancer. Recent studies have shown that the low-density lipoprotein receptor-related protein 1 (LRP-1) receptor has a significant impact on the development of breast cancer. In order to facilitate the identification of biomarkers, we have created a chimeric peptide that has been proven to bind successfully to the LRP-1 receptor.

**Methods:**

To identify biomarkers, we utilized advanced computational methods to conduct a meta-analysis of microarray data. Specifically, the g:Profiler and eXpression2Kinases (X2K) databases were utilized to identify gene ontologies and transcription factors. We then used the Human Protein Atlas to identify and assess crucial gene expressions.

**Results:**

Our results demonstrated that nucleolar and spindle-associated protein 1 (NUSAP1), melatonin receptor 1A (MELT), and cyclin-dependent kinase 1 (CDK1) are three hub genes that play pivotal roles in the pathogenesis of breast cancer.

**Conclusions:**

The research findings provide a deeper understanding of the molecular mechanisms involved in developing breast cancer. These findings have significant implications for developing novel therapies and diagnostics for this disease.

## 1. Background

Breast cancer is a significant public health issue that affects millions of women worldwide. Despite significant advances in early detection and treatment, breast cancer remains the leading cause of cancer-related deaths in women. Worldwide, 2.3 million new breast cancer cases were discovered in 2020, and 685,000 women died from the illness (1). The burden of breast cancer is particularly high in low- and middle-income countries, where access to timely and effective screening, diagnosis, and treatment is often limited. Therefore, continued research efforts are necessary to understand better the underlying mechanisms of breast cancer development and progression and to develop more effective strategies for prevention, diagnosis, and treatment (2). Chemotherapy and radiotherapy remain the mainstay of treatment for breast cancer patients. However, these treatments often lead to severe side effects, and the development of drug resistance is a major obstacle to successful cancer treatment. Therefore, novel and effective therapeutic strategies are urgently needed. Due to its great specificity and minimal side effects, peptide-based therapy has become a viable strategy for the targeted treatment of cancer.

Several studies have shown the potential of peptides as anticancer agents through different mechanisms, such as apoptosis induction, inhibition of angiogenesis, and modulation of the immune response (3). The design and development of peptide-based drugs for cancer treatment require a deep understanding of the molecular mechanisms underlying cancer progression and the identification of suitable targets. Molecular biology and bioinformatics advances have facilitated identifying novel targets and designing effective peptides. For example, developing peptide-based medicines with high affinity and specificity for cancer cells has been facilitated by identifying particular cell surface receptors and their associated ligands. In addition, computational tools such as molecular docking and molecular dynamics simulations have enabled the rational design of peptides with improved binding affinity and stability. Therefore, integrating experimental and computational approaches is crucial for developing effective peptide-based drugs for cancer therapy (4). One of the major challenges in breast cancer treatment is the development of drug resistance, which limits the efficacy of chemotherapy and targeted therapies. Identifying reliable biomarkers for breast cancer is essential for early detection, accurate diagnosis, and effective treatment. With the advancement of high-throughput technologies in genomics, transcriptomics, proteomics, and metabolomics, a large amount of data has been generated and made available to the research community. Bioinformatics approaches, including data mining, machine learning, and network analysis, have been used to analyze and integrate these data, leading to the discovery of novel breast cancer biomarkers. In this regard, several studies have reported the potential of different biomolecules, such as non-coding RNAs and proteins, as reliable biomarkers for breast cancer diagnosis and prognosis (5, 6).

**Figure 1.**
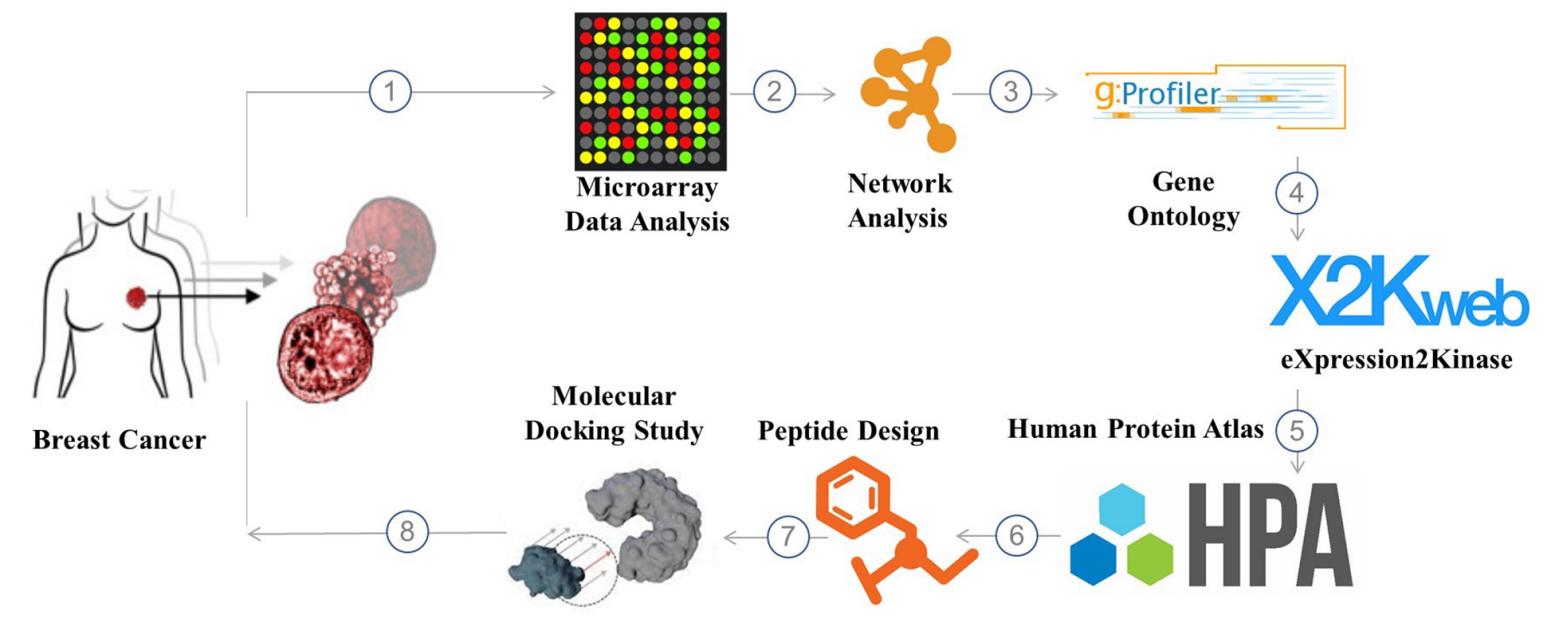
The schematic illustration of different steps of this work

Herein, an in-silico study was performed to uncover promising avenues for fighting breast cancer using a unique chimeric peptide. To generate the CSRLSLPGSSSKpalmSSS chimeric peptide, the SSSKpalmSSS heptapeptide (7) and the matrix metalloproteinase-9 (MMP-9) substrate enzyme sequence (CSRLSLPG) (8, 9) were merged. After exposure to an overexpressed quantity of MMP-9, the peptide (CSRLSLPGSSSKpalmSSS) was cleaved from the altered cleavage site in the tumor microenvironment (TME). According to simulation results, the remaining ten amino acid sequences (CSRLSLPGSS) that we call SRL-2 in this study displayed a significant affinity for the low-density lipoprotein receptor-related protein (LRP-1) in breast cancer. The discovery of these three key biomarkers: nucleolar and spindle-associated protein 1 (NUSAP1), melatonin receptor 1A (MELT), and cyclin-dependent kinase 1 (CDK1), and the potential use of a chimeric peptide in drug delivery represents a significant step forward in our understanding and management of breast cancer. These findings provide a solid foundation for developing new and effective diagnostic and therapeutic strategies, which can profoundly impact the lives of those affected by this disease. The schematic illustration of the different steps of this work is shown in Figure 1.

## 2. Methods

### 2.1. Microarray Data and Gene Expression Profile Analysis

The Gene Expression Omnibus (GEO) database of the National Center for Biotechnology Information (NCBI) supports community-derived reporting standards; GEO (10), for example, can identify the presence of many crucial study components like raw data, processed data, and descriptive metadata. The microarray gene expression profile of primary breast cancer (PBC) data was acquired from the NCBI GEO database (http://www.ncbi.nlm.nih.gov/geo). GEO was employed to retrieve the gene expression profile dataset with access number GSE205185. The Agilent-072363 SurePrint G3 Human GE v3 8x60K Microarray 039494 (Probe Name Version) was used, which consisted of 17 PBC samples and five normal breast cancer samples.

### 2.2. Protein-Protein Interaction Network Analysis

In order to incorporate all of the known and anticipated links between proteins, including both their physical interactions and their functional correlations, the STRING database was created. STRING gathers and analyzes data from various sources, some listed below, to accomplish this purpose. Using co-expression and preserved genomic context, computational interaction predictions, databases of annotated complexes and pathways, interaction experiments, automated text mining of scientific literature, and systematic transmission of interaction evidence from one organism to another are just a few methods used to study interactions (11). Version 11.5 of STRING (https://string-db.org), a database search engine for interacting genes, incorporates both known and anticipated protein-protein interaction (PPI) and can be used to forecast functional interactions between differentially expressed genes (DEGs) (high confidence score of 0.700 was set as the cut-off criteria to construct a PPI network). Lastly, hub genes were discovered using the cytoHubba (version 0.1) plug-in of the Cytoscape software (version 3.9.1; www.cytoscape.org (12).

### 2.3. Functional Enrichment Analysis and Conversions of Gene Lists (g:Profiler)

g:Profiler is a publicly accessible web server found at http://biit.cs.ut.ee/gprofiler/. It is used to characterize and manipulate gene lists obtained through the analysis of high-throughput genomic data. The functional enrichment analysis was conducted utilizing g:Profiler (version e107 eg54 p17 bf42210) with the g: SCS multiple testing correction approach and a significance level of 0.05 (13).

### 2.4. Detection of Transcription Factors (TFs) and Kinases

We searched the ChIP enrichment analysis (ChEA) database for transcription factors (TFs) that may regulate the expression of genes associated with PBT. Eukaryotic TFs, consensus bond sequences (positional weight matrices), empirically validated bond areas, and regulated genes are all covered by the ChEA database. Also, the putative TFs, protein complexes, and protein kinases that are most likely to be in charge of the observed alterations in PBT transcriptomes were identified and ranked using eXpression2Kinases (X2K) (https://amp.pharm.mssm.edu/X2K/) (14).

### 2.5. Hub Gene Selection and Validation in the Human Protein Atlas

The Human Protein Atlas database (https://www.proteinatlas.org/), a Swedish-based program launched in 2003 to survey all human proteins in cells, tissues, and organs by integrating various omics technologies, was used to identify the hub gene expression levels between cancer patients and healthy controls. Additionally, we visualized the expression of key hub genes in PBC samples and normal ovarian surface epithelia using boxplots and Gene Expression Profiling Interactive Analysis, a recently developed interactive web server for analysis of the RNA sequencing expression data of 9,736 tumors and 8587 normal samples from the TCGA and the GTEx projects (15).

### 2.6. Structure Preparation

Using the protein preparation protocol in the Pymol software package, we optimized the structure of LRP-1 (PDB code: 2KNX) that we had previously downloaded from the protein data bank (PDB).

### 2.7. Methodology for Peptide Prediction

A peptide candidate’s three-dimensional structure was analyzed using a de novo peptide structure prediction server called PEP-FOLD3, a technique for predicting de novo peptide structures based on amino acid sequences.

### 2.8. Molecular Docking

CABS-dock was used so that we could carry out the molecular docking process. In a simpler description, this server facilitates the docking of flexible proteins and peptides. The PDBsum server investigated the following results. The server is an online database containing statistics and docking computation results (www.ebi.ac.uk).

## 3. Results

### 3.1. Exploring Receptor Activity and Unveiling Peptide Candidate Structures

**Figure 2.**
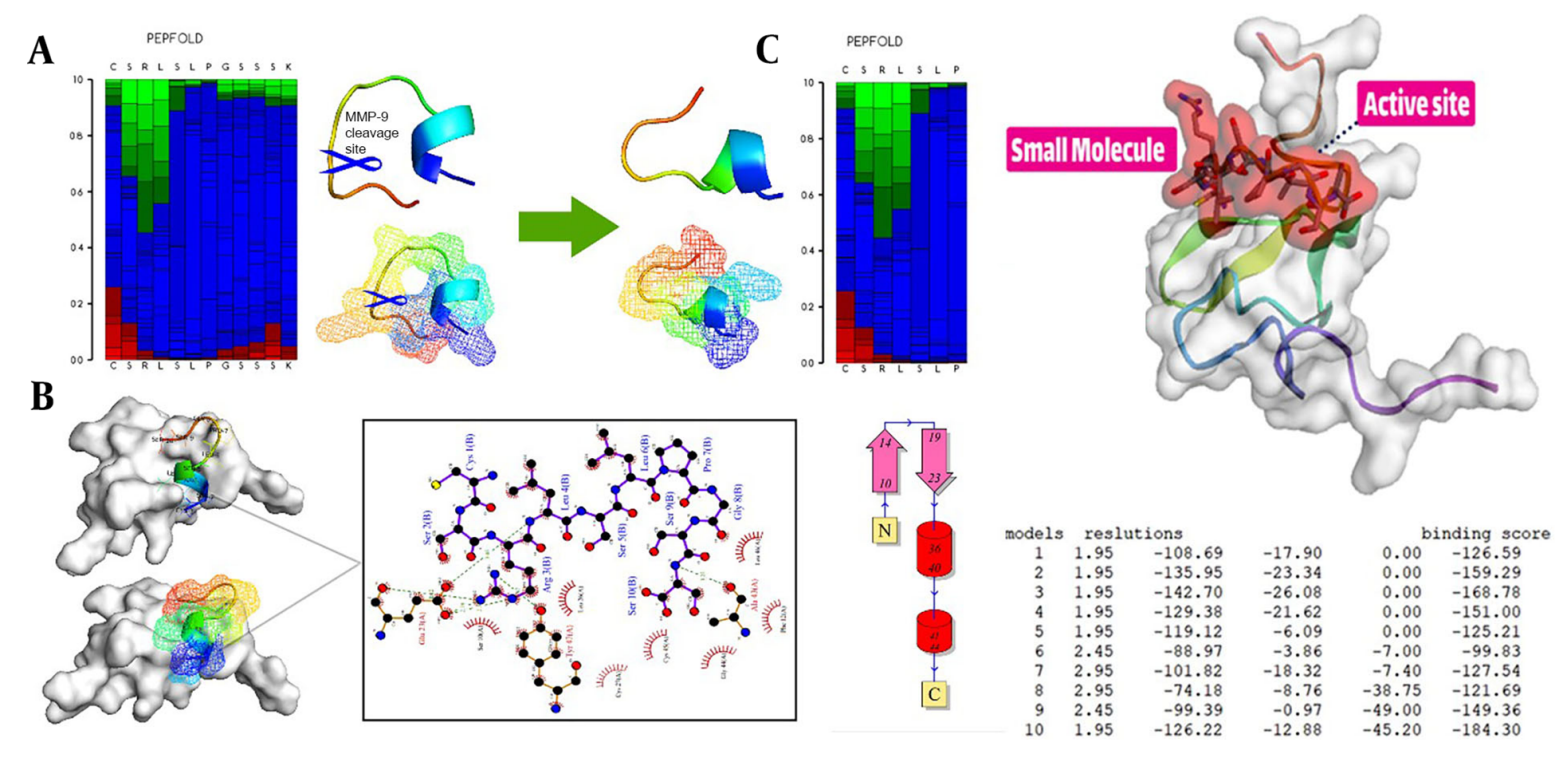
Peptide design and protein-peptide docking using CABSDock server. A, The results of detecting the primary structure of a three-dimensional structure using PEPFold3 are as follows: Red denotes helical states, green represents extended states, and blue represents all other states; B, When the MMP-9 enzyme cleaves the chimeric peptide, the chimeric peptide maintains its function and actions; B and C, Molecular docking revealed that the chimeric peptide had a particular affinity for the active site of the LRP-1 protein. A computer program called LIGPlot creates 2-D schematics of protein-ligand interactions using the Protein Data Bank’s common input files. The PDBsum resource, which offers a summary of the molecular structure, uses LIGPlot to produce graphics.

To begin the process, the receptor’s structure was meticulously scrutinized to identify any potential missing residues (PDB id 2KNX). Once the structural integrity and completeness of the protein were verified, it was forwarded to the PLIP server to obtain essential information on its active site. These fundamental steps are the cornerstone of in-silico research and demand high precision and proficiency from the researcher. The results suggest that our cleavable stealth chimera peptide (SRL-2) could potentially serve as a targeted therapy for breast cancer by selectively binding to LRP-1 in the TME and evading immune detection due to its antifouling properties. However, further experimental validation is required to confirm the efficacy and safety of our cleavable stealth chimera peptide. The three-dimensional structure of a potential peptide was discovered using the PepFold3 service. The primary structure is determined when the chimeric peptide is in its primary state and is unmodified by the MMP-9 enzyme. In the second stage, the structure is determined when the peptide is cleaved via the MMP-9 enzyme. After the enzyme breaks the peptide at a specific site, the new structure is folded and compared to the original structure (Figure 2A and B).

### 3.2. Molecular Docking Study

With protein-peptide docking, the HDOCK server has divulged the mesmerizing fact that the peptide contender exhibits an extraordinary affinity for the receptor. Moreover, by scrutinizing the peptide candidate image via the Discovery Studio Visualizer tool, the receptor cavity was astutely unearthed in the active site of the receptor, and as the cherry on top, the PDBsum server came in handy to demarcate the chemical boundaries and compile a register of amino acids implicated in the interactions, which were further investigated with the aid of the Ligplot (Figure 2B and C).

### 3.3. Identification of DEGs

**Figure 3.**
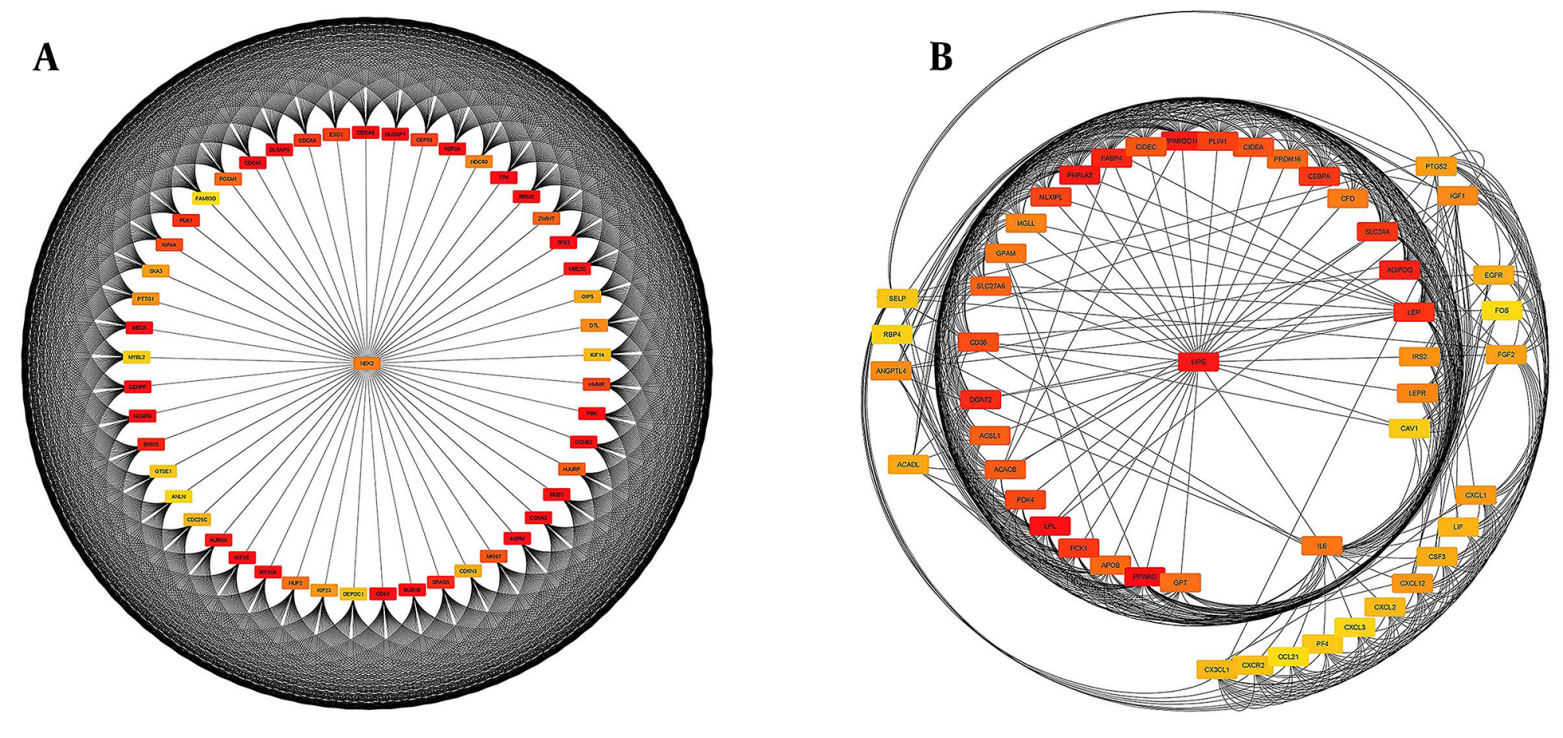
Cytoscape software displayed protein-protein interaction (PPI) network analysis. A, The upregulated gene network of 50 genes is essential for proper cellular function and regulation; B, The downregulated gene network of 50 genes can lead to disrupted cellular processes and potential disease development.

**Figure 4.**
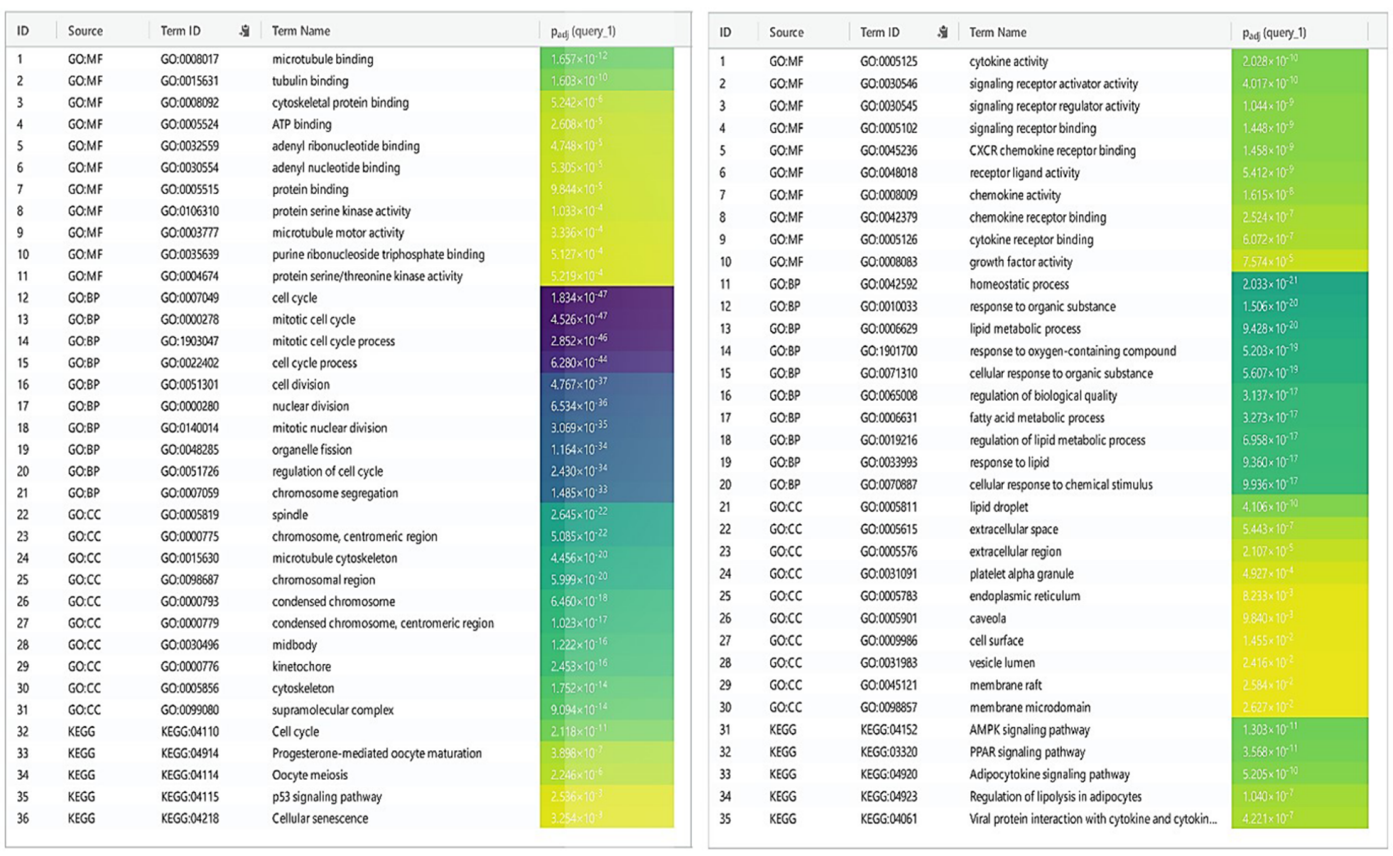
g:Profiler, a web server for functional enrichment analysis and gene ontology. Gene ontology analysis of upregulated genes can provide insights into the biological processes and pathways potentially involved in a cellular phenotype or disease. For a downregulated gene network: Gene ontology analysis can uncover the potential molecular mechanisms and pathways responsible for the observed cellular dysfunction or disease state by examining the functional annotation of the downregulated genes.

**Figure 5.**
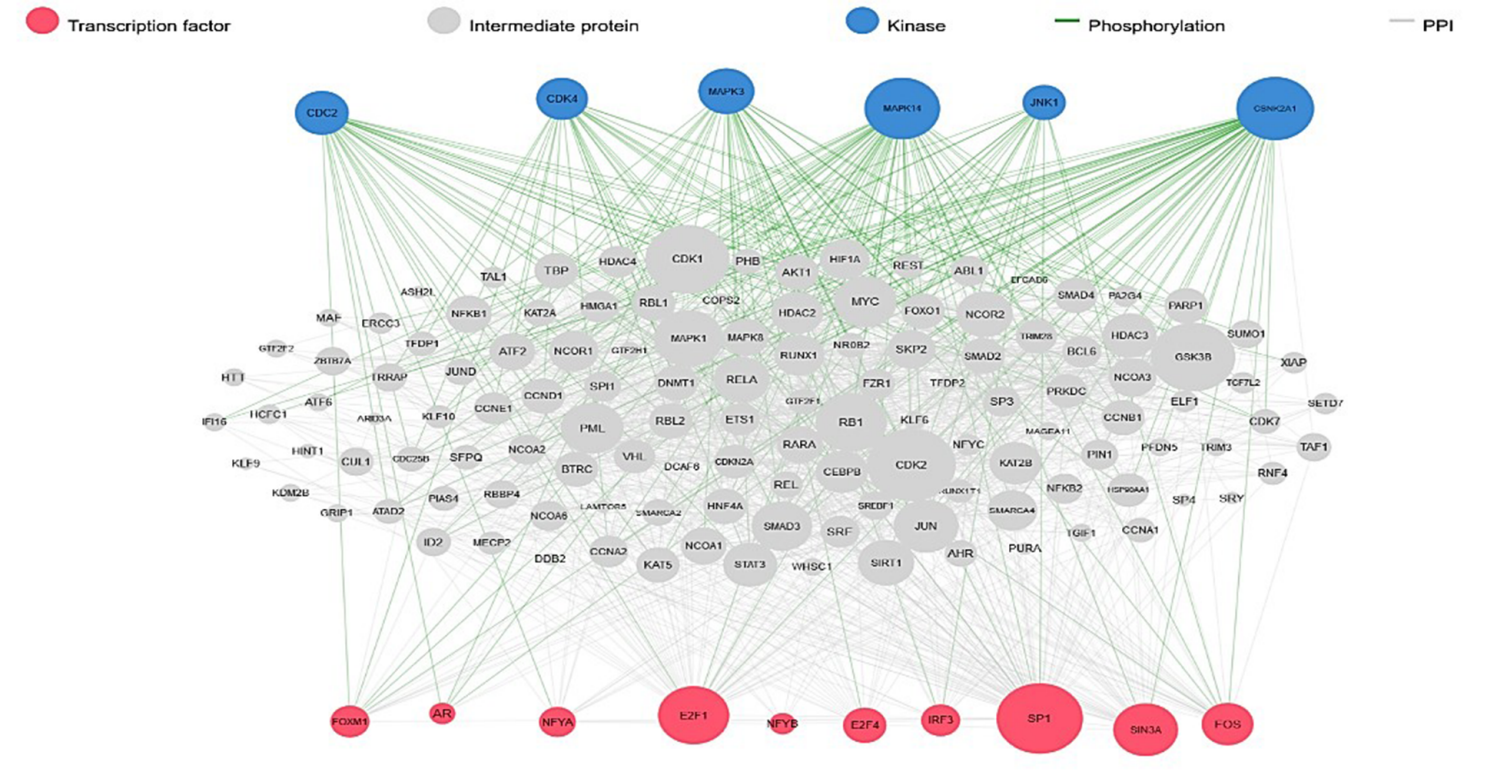
Transcription factor (TF) and kinases prediction by using eXpression2Kinases (X2K) server. Analysis of transcription factors and kinases as biomarkers can provide valuable insights into the signaling pathways and cellular processes that underlie various diseases, aiding in disease diagnosis, prognosis, and targeted therapy development.

**Figure 6.**
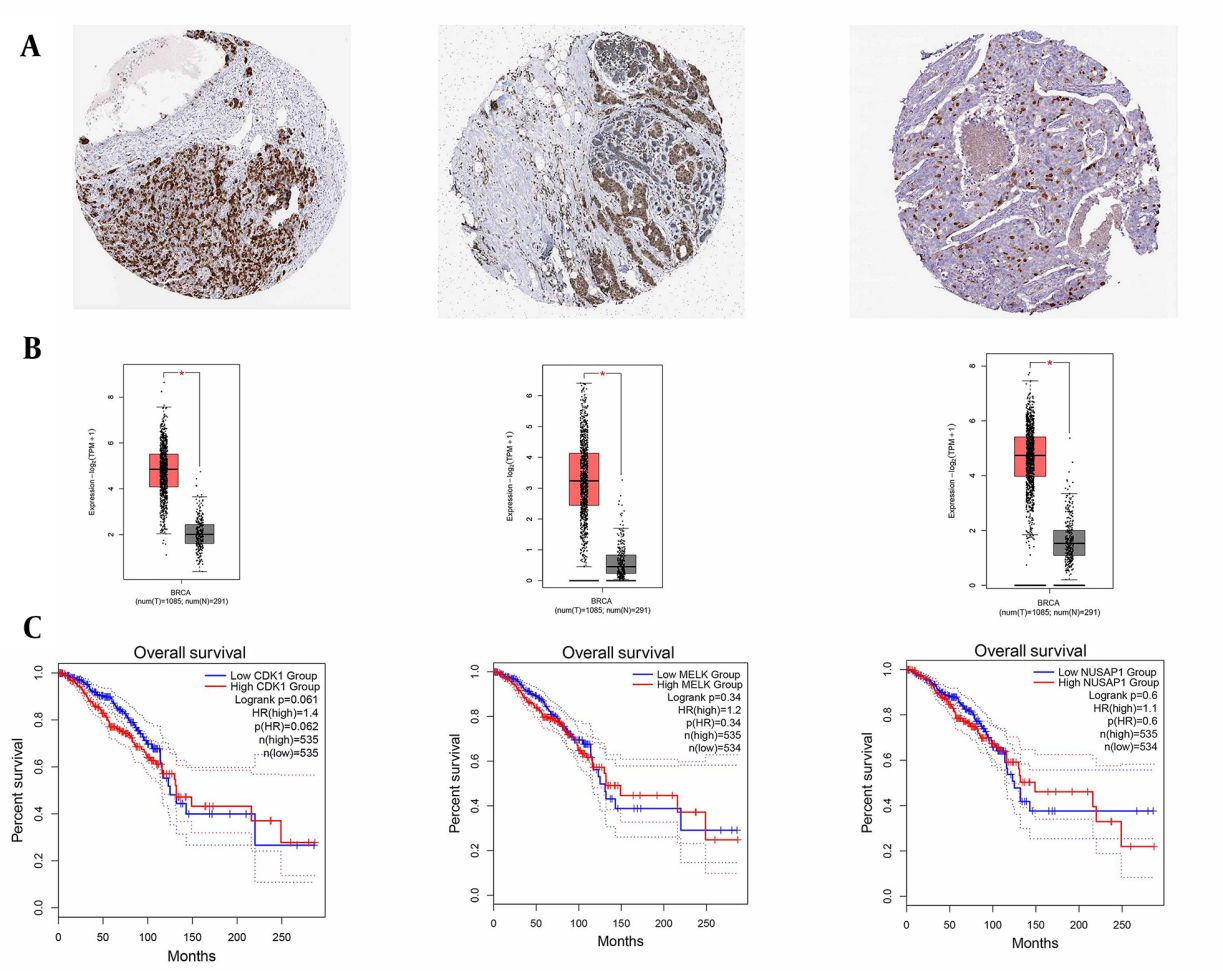
Human protein atlas and overall survival. A and B, It illustrated the increased expression levels of cyclin-dependent kinase 1 (CDK1), maternal embryonic leucine zipper kinase (MELK), and nucleolar and spindle-associated protein 1 (NUSAP1) genes from left to right in breast cancer cells; C, The server overall survival displayed the increased expression levels of CDK1, MELK, and NUSAP1 genes from left to right in breast cancer cells. Its main purpose is to provide information on the relationship between gene overexpression and overall survival compared to the control. In essence, the Overall Survival server analyzes clinical and genetic data from different patients to understand the correlation between gene expression and the overall survival time of breast cancer patients.

DEGs were identified in 17 PBC samples and five normal breast tissue (NBC) samples. When using the Limma R tool to find DEGs, a statistically significant criterion of P-value < 0.05 and |LogFC| > 2 was utilized (Figure 3A). Using the STRING database, interactions between up-and down-regulated genes were examined, revealing 453 PBC-related genes and 1040 NBC-related genes. Hub genes were identified using Cytoscape software v3.9.1 (cytoHubba plug-in). Finally, we utilized the Cytoscape plug-in Cytohobba and configured the parameters to identify the top 50 genes with maximal clique centrality to generate PBC and NBC (Figure 3B). To gain insights into the functional roles of the DEGs identified in our study, we performed gene ontology (GO) analysis using the g:Profiler server. We submitted a list of DEGs’ top 50 hub genes (up and down genes) and selected the “hsapiens” database as our reference organism. The enrichment analysis was performed after multiple test corrections with the Benjamini-Hochberg technique and with a significance level of P-value < 0.05. The GO data were divided into three main categories: Biological processes, cellular components, and molecular function. The output provided us with a comprehensive list of enriched terms, allowing us to gain a better understanding of the biological processes and functions associated with our DEGs (Figure 4). In the realm of cancer development, transcription factors and kinases are two prominent classes of molecular players that hold significant influence. Their control over gene expression and cellular signaling pathways confers them with the power to direct critical cellular processes. Our study has made groundbreaking progress in identifying key transcription factors, such as SP1, E2F1, and SIN3A, as well as kinases, including MAPK14, CSNK2A1, and CDC2, whose role in breast cancer progression is paramount (Figure 5). Recently, several studies have investigated the prognostic value of various genes in breast cancer, including NUSAP1, maternal embryonic leucine zipper kinase (MELK), and CDK1. These genes are linked to poor overall survival in breast cancer patients and have been demonstrated to have significant roles in tumor progression. NUSAP1 is a gene that encodes a protein involved in DNA damage response and repair. High expression of NUSAP1 has been linked to more aggressive breast cancer phenotypes and worse overall survival. Like MELK, NUSAP1 is a protein kinase that promotes cell proliferation and survival, and its overexpression in breast cancer patients has been linked to a poor prognosis. CDK1, a cell cycle regulator, has also been implicated in breast cancer progression and is associated with decreased overall survival (Figure 6). Overall survival analysis of NUSAP1, MELK, and CDK1 in breast cancer patients has provided valuable insights into the underlying mechanisms of tumor progression. It highlights the importance of personalized treatment strategies. Targeting these genes and their associated pathways may hold promise for improving clinical outcomes in breast cancer patients. However, further research is needed to fully understand the potential of these genes as prognostic markers and therapeutic targets in breast cancer.

## 4. Discussion

Breast cancer remains one of the most prevalent global public health challenges. It requires persistent research efforts to devise more efficient prevention, diagnosis, and treatment strategies. Recently, peptide-based therapy has emerged as a promising approach for targeted cancer treatment. Integration of structural and computational methods is crucial for developing effective peptide-based drugs (16). Moreover, bioinformatics approaches can aid in identifying dependable biomarkers for breast cancer, resulting in timely detection, precise diagnosis, and efficient treatment (17). This highlights the requirement for novel therapeutic approaches founded on thoroughly comprehending the molecular pathways driving breast cancer’s onset, recurrence, and metastasis. By examining the gene expression profile of these tumors, particularly at the primary stage, it may be feasible to identify the mechanisms underlying the genesis and progression of breast cancer. We conducted a comprehensive analysis to identify elevated genetic variables during the early stages of breast cancer formation. This was done to gain a better understanding of the intricate biological mechanisms that contribute to the development of breast cancer. Our study sought to make clear the mechanisms by which these genes may contribute to tumor initiation and progression, with the ultimate goal of informing the development of novel therapeutic strategies for this devastating disease. We used a variety of bioinformatics analyses to address the following issues: (1) Which signaling pathways are the most important for breast cancer? (2) What hub genes based on breast cancer were created in the PPI network? (3) Which kinases and TFs control the expression of breast cancer? (4) How did peptide-based therapy become a potential strategy for treating cancer with precision? The g:Profiler database was used to examine signaling pathways, and it was discovered that breast cancer-associated genes have important roles in cellular response. Notably, the p53 signaling pathway is critical for maintaining genomic stability and preventing tumor formation and progression. This pathway encodes the p53 gene, often called the “guardian of the genome.” It acts as a transcription factor to regulate the expression of several target genes involved in aging, DNA repair, cell cycle arrest, and apoptosis. The activation of the p53 pathway occurs due to various cellular stresses, such as DNA damage, hypoxia, and oncogene activation. As p53 is activated, it goes through post-translational changes that cause it to become stabilized and activated as a transcription factor. The downstream targets of p53 activation eventually result in the removal of damaged cells or the restoration of genomic stability, thereby preventing cancer development (18). A fuller understanding of the signaling pathways and breast cancer components could result in efficient treatment plans that inhibit cancer cell proliferation with few side effects and high chemotherapy sensitivity. Furthermore, our research has shown that MELK, NUSAP1, and CDK1 are crucial hub genes that are overexpressed in breast cancer and have potential roles as biomarkers. These genes have been implicated in several key processes in breast cancer tumorigenesis, including cell proliferation, DNA replication, and cell cycle regulation. The upregulation of these genes in breast cancer raises the possibility that they have a major impact on the onset and spread of breast cancer and may serve as potential targets for developing novel therapeutic strategies (19). Our research team extensively experimented to develop a novel approach for drug delivery to breast cancer cells. After exploring several possibilities, we settled on the use of a chimeric peptide. This type of peptide is made by combining two peptides with different functions into a single molecule. We utilized a structural-based peptide design technique to create a peptide that could effectively target breast cancer cells. Our designed chimeric peptide could bind successfully to the LRP-1 receptor, which is overexpressed in breast cancer cells. We also performed molecular docking studies to confirm the efficacy of our peptide in drug delivery.

### 4.1. Conclusions

In conclusion, our meta-analysis of the literature suggests that incorporating chimeric peptides, specifically designed to interact with the LRP-1, holds promise as a targeted approach for drug delivery in breast cancer treatment. Utilizing these chimeric peptides, structure-based peptide design, and molecular docking techniques provides a potential avenue for developing personalized and effective therapeutic strategies. Furthermore, identifying and validating biomarkers associated with LRP-1-mediated drug uptake and response could serve as valuable tools for patient stratification and treatment optimization. Further experimental studies are warranted to validate the clinical utility of chimeric peptides and their potential as biomarkers in breast cancer management.
